# Pandemic (H1N1) 2009 Transmission during Presymptomatic Phase, Japan

**DOI:** 10.3201/eid1709.101411

**Published:** 2011-09

**Authors:** Yoshiaki Gu, Nobuhiro Komiya, Hajime Kamiya, Yoshinori Yasui, Kiyosu Taniguchi, Nobuhiko Okabe

**Affiliations:** Author affiliation: National Institute of Infectious Diseases, Tokyo, Japan

**Keywords:** Influenza, human, influenza A virus, viruses, H1N1 subtype, pandemic (H1N1) 2009, disease outbreaks, infectious disease transmission, infectious disease incubation period, contact tracing, dispatch

## Abstract

During an epidemiologic investigation of pandemic influenza (H1N1) 2009 virus infection in May 2009 in Osaka, Japan, we found 3 clusters in which virus transmission occurred during the presymptomatic phase. This finding has public health implications because it indicates that viral transmission in communities cannot be prevented solely by isolating symptomatic case-patients.

The first indigenous cases of pandemic (H1N1) 2009 in Japan were detected in Kobe City (*1*) and Osaka Prefecture on May 16, 2009. In response to the outbreak, the National Institute of Infectious Diseases, Infectious Diseases Surveillance Center, and its Field Epidemiology Training Program began epidemiologic investigations in both areas. Clinical manifestations of these infections were described in 2 previous articles (*2**,**3*).

In general, pandemic influenza (H1N1) 2009 virus is considered to be infectious during patients’ presymptomatic phase (*4**,**5*). However, to our knowledge, no epidemiologic studies about infectiousness during the presymptomatic phase have been reported. The aim of this study was to provide scientific evidence to ascertain the infectious period of pandemic influenza (H1N1) 2009 through epidemiologic investigation in Osaka, Japan.

## The Study

We began an epidemiologic investigation in Osaka on May 17, 2009, and conducted face-to-face interviews. Thirty-six confirmed cases had occurred by May 22. The definition of a confirmed case-patient was a person with influenza-like illness (ILI) and laboratory confirmation of pandemic (H1N1) 2009 virus infection by real-time reverse transcription PCR. ILI was defined as the presence of a fever (>38.0°C) and acute respiratory symptoms (cough or sore throat). Local public health staff also conducted face-to-face or telephone interviews, or both, with other patients and persons in close contact with the case-patients until the end of May, when the local epidemic appeared to wane (*1**,**6*). Interviews were conducted by using a standard questionnaire to collect data on patient demographics, clinical course of illness, patient behavior, and history of patient contacts. By analyzing these data and careful investigation of contact history and epidemiologic links, we made a transmission tree. During the epidemiologic investigation, we found 3 clusters in which disease transmission could have occurred before symptom onset in the index case-patients (Figure).

**Figure:**
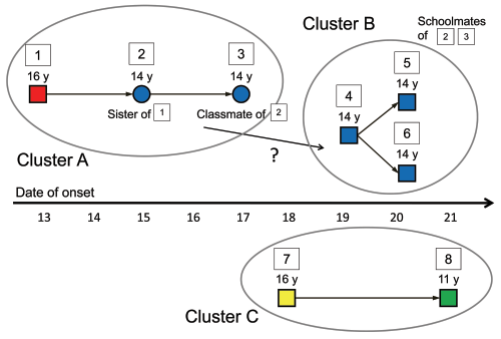
Three clusters of pandemic (H1N1) 2009 presymptomatic transmission in May 2009 in Osaka, Japan. All cases were confirmed as pandemic (H1N1) 2009 virus infection by real-time reverse transcription PCR. Squares indicate male case-patients, and circles indicate female case-patients. Colors of the squares and circles denote the similar or different schools the students attended.

The first outbreak in Osaka occurred in 1 high school, and no community transmission had been verified in mid-May. Case-patient 1, a student at the high school, had symptoms on May 13. Case-patient 2, the sister of case-patient 1, had no symptoms while she was at school, according to her answer on the self-report survey as well as face-to-face interview carried out with a classmate who sat right behind her (case-patient 3). Symptoms developed in the evening of May 15 after she returned home, and the school was closed proactively the following day. She later received a diagnosis of infection with the virus. No other students or staff had ILI symptoms at that time. For case-patient 3, who sat immediately behind case-patient 2 in class and chatted with her, influenza-related symptoms developed on May 17. Our investigation suggested that transmission of infection from case-patient 2 to case-patient 3 occurred during the presymptomatic phase of infection in case-patient 2.

Case-patients 4, 5, and 6 were previously healthy boys and schoolmates of case-patients 2 and 3. They did not have contact with anyone with ILI symptoms at school or elsewhere. They played video games together in a small room on May 18 for several hours. They took turns using the same game consoles and chatted together. Case-patient 4 became febrile on the morning of May 19. He may have been infected by case-patient 2 at school, but we cannot confirm this. Case-patients 5 and 6 became febrile on May 20. They had no opportunity to be in contact with symptomatic persons other than case-patient 4. Thus, we concluded that case-patients 5 and 6 were likely infected by case-patient 4 before symptom onset. Even if transmission from case-patient 2 were the reason that case-patients 5 and 6 became ill, transmission from a presymptomatic person likely occurred because case-patient 2 would have been asymptomatic when she met case-patients 5 or 6, or both, at school.

Case-patient 7 attended a different high school than case-patients 1–6. Case-patient 8 was an elementary school pupil who lived in a different region from case-patients 1–7. No cases of pandemic (H1N1) 2009 had been reported in the area where he lived, including at his school, in mid-May.

Investigation results indicate that case-patient 7 was infected by a classmate other than case-patients 1–8 around May 16; this classmate was infected by a student at the same high school that case-patient 1 attended. On May 17, ≈20 persons, including case-patients 7 and 8, went on a 1-day trip to Okayama Prefecture, which is ≈180 km west of Osaka. The trip took ≈3 hours by train, which was not crowded. No positive results were reported by active surveillance for ILI in Okayama in mid-May. Case-patients 7 and 8 stayed close together during the trip. In the evening of May 18, case-patient 7 had a sore throat and cough, and he became febrile on May 19. Case-patient 8 had symptoms on May 21, 3 days after case-patient 7 exhibited symptoms. No one around them had respiratory symptoms during the trip. The trip was the only opportunity for case-patient 8 to have been exposed to the virus. This indicates that transmission from case-patient 7 to case-patient 8 occurred on the day before symptom onset.

## Conclusions

Our epidemiologic investigation results indicate that pandemic influenza virus (H1N1) 2009 is infectious during the presymptomatic phase. Our investigation was conducted in the early phase of the outbreak in Japan. During this period, almost all persons with reported cases in Osaka were students and their close contacts (*1*). Surveillance data from the Osaka Prefectural Institute of Public Health supported this finding. The institute had been conducting active surveillance for patients with ILI and suspected pandemic (H1N1) 2009 virus infection since April 2009 (*7*). Since mid-June, many community-transmitted cases had been reported in Japan, thereby limiting the ability to find single exposure cases. Furthermore, most cases were no longer laboratory confirmed. As such, our results are valuable in terms of collecting information before the community spread of the virus.

The mode of transmission of pandemic (H1N1) 2009 is considered to be similar to that of seasonal influenza. The infectious period of seasonal influenza is measured from 1 day before symptom onset to 5–7 days after onset or until symptoms resolve; this period is mainly based on experimental data on viral shedding (*4**,**5*). Regarding seasonal influenza, we found only 1 report documenting that a transmission took place during the presymptomatic phase, but the index case-patient of the report could have been in a “prodromal,” not “presymptomatic,” state because this case-patient did not feel completely well when the transmission took place (*8*).

Our epidemiologic results provide useful clues for understanding the transmission of pandemic (H1N1) 2009 virus. This finding of presymptomatic transmission has critical implications for public health because it indicates that viral transmission in communities cannot be completely prevented solely by isolating symptomatic case-patients.

Two possible limitations of this report are the presence of unidentified case-patients and asymptomatic infected persons because our epidemiologic investigation was based on interviews with symptomatic patients and their close contacts. However, we could not find any cases that suggested infection from these kinds of cases during the early phase of the outbreak in Japan. Additionally, social attention was very high during the period.

In conclusion, our epidemiologic investigation results suggest that pandemic (H1N1) 2009 is probably infectious during the presymptomatic period, at least 1 day before symptom onset. Our results are consistent with the description of the infectious period of the virus.
